# An Image-Based Augmented Reality System for Achieving Accurate Bone Resection in Total Knee Arthroplasty

**DOI:** 10.7759/cureus.58281

**Published:** 2024-04-15

**Authors:** Hyoung-Taek Hong, Yong-Gon Koh, Byung Woo Cho, Hyuck Min Kwon, Kwan Kyu Park, Kyoung-Tak Kang

**Affiliations:** 1 Skyve R&D LAB, Skyve Co. LTD., Seoul, KOR; 2 Joint Reconstruction Center, Department of Orthopedic Surgery, Yonsei Sarang Hospital, Seoul, KOR; 3 Department of Orthopedic Surgery, Severance Hospital, Yonsei University College of Medicine, Seoul, KOR; 4 Department of Orthopedic Surgery, Yonsei University College of Medicine, Seoul, KOR; 5 Mechanical Engineering, Yonsei University, Seoul, KOR

**Keywords:** healthcare tech contest, navigation, total knee arthroplasty, augmented reality, computer assisted surgery, image based

## Abstract

Background and objective

With the steady advancement of computer-assisted surgical techniques, the importance of assessing and researching technology related to total knee arthroplasty (TKA) procedures has increased. Augmented reality (AR), a recently proposed next-generation technology, is expected to enhance the precision of orthopedic surgery by providing a more efficient and cost-effective approach. However, the accuracy of image-based AR in TKA surgery has not been established. Therefore, this study aimed to determine whether accurate bone resection can be achieved in TKA surgery using image-based AR.

Methods

In this study, we replaced traditional CT imaging and reconstructions for creating a bone 3D model by direct 3D scanning of the femur and tibia. The preoperative planning involved identifying anatomical landmarks and determining the surgical details. During surgery, markers were employed to create a local coordinate system for an AR-assisted surgical system using a Polaris camera. This approach helped minimize discrepancies between the 3D model and actual positioning, ensuring accurate alignment.

Results

The AR-assisted surgery using the image method resulted in fewer errors [average error: 0.32 mm; standard deviation (SD): 0.143] between the bone resection depth of the preoperative surgical plan and the bone model test results.

Conclusions

Our findings demonstrated the accuracy of bone resectioning by using image-based AR-assisted navigation for TKA surgery. Image-based AR-assisted navigation in TKA surgery is a valuable tool not only for enhancing accuracy by using smart glasses and sensors but also for improving the efficiency of the procedure. Therefore, we anticipate that image-based AR-assisted navigation in TKA surgery will gain wide acceptance in practice.

## Introduction

Total knee arthroplasty (TKA) is an effective treatment method for alleviating severe knee osteoarthritis by reducing pain and enhancing joint function. TKA is used to improve a patient's quality of life and to reduce pain, and it has been associated with long-term implant survival, successful functional outcomes, and minimal complications [1,2]. The crucial determinants of successful and enduring TKA include the precise positioning of the implanted components and the appropriate alignment of the knee [3]. Innovative TKA technologies are regularly emerging, thereby helping to improve surgical outcomes, enhance patient safety, and increase surgical efficiency and cost-effectiveness [4]. The success in achieving these targets varies significantly among surgeons, and the adoption of computer-assisted technologies aims to shorten the learning curve and mitigate the impact of surgeon proficiency variability on surgical procedures [4].

Recent years have witnessed a renewed focus on augmented reality (AR) technology in this context, particularly in the field of orthopedic surgery to enhance TKA precision [5]. Unlike virtual reality (VR), AR augments the real-world scenario with virtual information, whereby the user is provided with a completely virtual environment [6,7]. The user's vision is augmented through a monitor-based display system, an optical see-through system, or a video see-through system [8]. During surgery, devices such as smart glasses are utilized to generate enhanced overlays of cut surfaces or axes, enabling surgical professionals to facilitate precise instrumentation [9,10]. Tsukada et al. conducted a study to assess the accuracy of the AR-KNEE system, an imageless navigation system that leverages AR technology for tibial bone resection in TKA [11]. Their study using sawbones suggested that the AR-KNEE system may offer reliable accuracy for coronal, sagittal, and rotational alignment during tibial bone resection in TKA [11]. They have since expanded their research to address femoral bone resection in TKA [12]. Meanwhile, Bennett et al. determined that AR navigation can achieve precise alignment in TKA with a low incidence of component malpositioning in the coronal plane [13]. However, the above-mentioned studies on TKA utilizing AR were based on imageless technology.

Although imageless technology-based AR surgery has demonstrated superior accuracy, it can be quite time-consuming, averaging 10-15 minutes per case, compared with image-based AR-assisted surgery, and necessitates a prolonged learning curve. This extended duration can be attributed to the fact that, unlike image-based methods where surgical planning is performed preoperatively, imageless technology-based computer-assisted surgery involves registering anatomic landmarks and conducting surgical planning during the procedure. In light of this, the objective of this study was to determine whether accurate bone resection can be achieved in TKA surgery by using image-based AR. We hypothesized that image-based AR in TKA could achieve precise bone resection.

## Materials and methods

2.1. Overview

**Figure 1:**
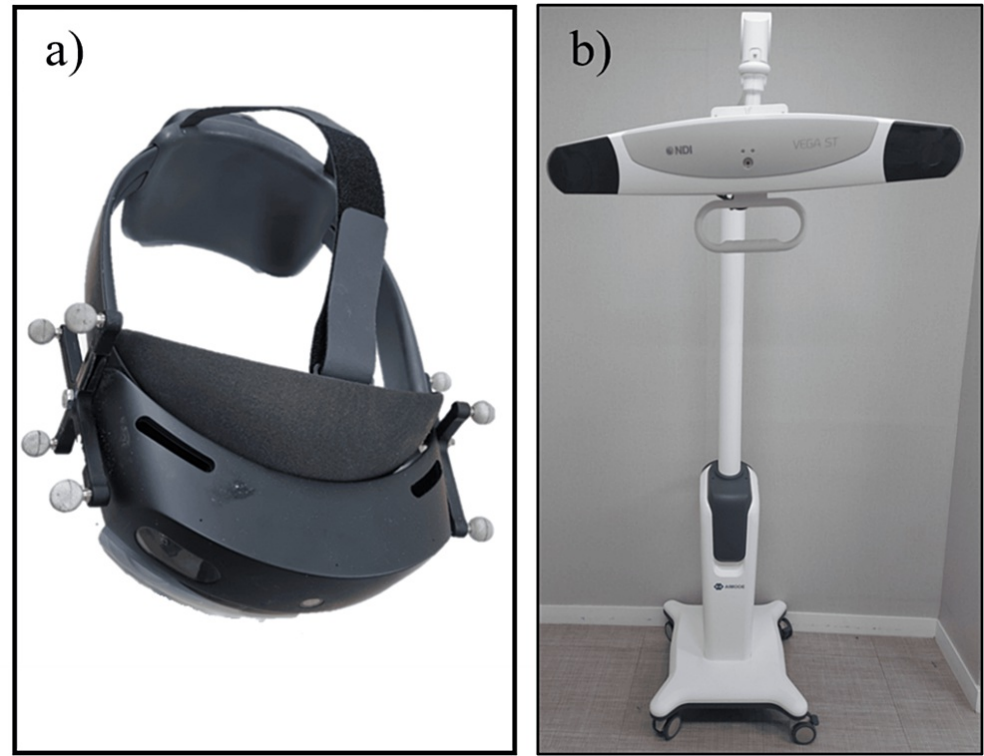
Components of the AR-assisted navigation system a) HoloLens 2 goggles. b) Polaris Vega optical tracking system AR: augmented reality

The AR-assisted navigation system (Sagarvision AR, Skyve Co. Ltd., Seoul, South Korea) primarily comprises an optical tracking system (Polaris Vega, Northern Digital Inc., Waterloo, Canada), and AR glasses (HoloLense2, Microsoft, Redmond, WA), as shown in Figure 1.

To build the software for the proposed system, a combination of various open-source libraries was utilized, including IGSTK12 for interfacing with the optical tracking system, OpenCV and ARToolKit for image processing, and OpenGL for rendering 3D anatomical objects onto the camera images. The position and orientation of the camera were tracked in real-time by monitoring the attached tracker. A precalibration process was performed to establish the transformation relationship between the tracker and the optical tracking system. Moreover, a paired-point registration method was employed to determine the transformation relationship between the world coordinate system and the dynamic reference frame coordinate system. To synchronize the virtual camera's position and orientation with the actual camera in real-time, the spatial coordinates of the camera were transformed into the world coordinate system. Ultimately, the projection of the virtual camera was aligned with that of the real camera through a calibration process.

2.2. Tracker-camera calibration for positional accuracy of the AR-assisted navigation system

**Figure 2:**
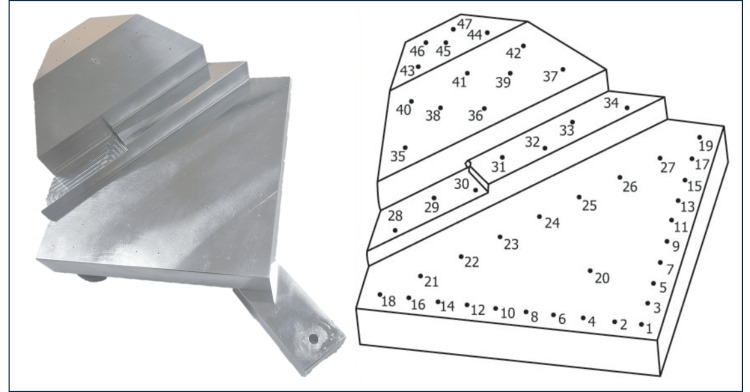
Phantom for assessing tracking accuracy in computer-aided surgery systems

A phantom featuring specific divots at known locations was securely mounted using a rigid, multi-axis repositionable arm, as shown in Figure 2. A computer system equipped with suitable software was connected to a tracker and a nearby viewing screen to provide real-time positional data for operator reference. The phantom was designed per ASTM F2554-22 [14], the standard for assessing tracking accuracy in computer-aided surgery systems. The evaluation was conducted per ASTM F2554-22 [14], including both single-point and multi-point tests within the tracker's operational volume. In these tests, data were captured through screen recordings showing the pointer's real-time positional data.

2.2.1. Single-Point Accuracy Test

In the single-point accuracy test, the phantom was treated as a part of the patient's anatomy. The test involved repeatedly measuring a specific point on the phantom (central divot, #20 in Figure 2) to assess the accuracy of the measurements concerning the phantom's actual position within its coordinate system. This process was repeated 20 times to establish bias, which was calculated as the difference between the average of the measured points and the actual location of the central divot. The average error vector was determined by summing up all error vectors and dividing the resultant vector length by the number of vectors. The outcomes included the average error vector and the length of the longest error vector. To gauge precision, we calculated the average location of all measurements to determine the system's best estimate of the central divot's position [14]. Then, we calculated each measurement distance from this average point and identified the root mean square (RMS) of these distances and their maximum.

2.2.2. Point-to-Point Accuracy Test

The point-to-point test was conducted to assess the accuracy of the measured distances between various points on the phantom. At least 20 measurements, including divot #30 (shown in Figure 2), were performed, ensuring a minimum of two points per plane (with four on the slanted plane). For each pair of measured points, the distance was calculated, resulting in 190 distances for 20 points. The accuracy was determined by comparing these measured distances to the ground truth distances obtained from the phantom's coordinate measuring machine (CMM) measurements [14]. The discrepancies between these sets of distances were recorded, and the maximum error and the RMS of these errors were noted.

2.3. Patient registration and AR-assisted surgery

2.3.1. Preoperation

**Figure 3:**
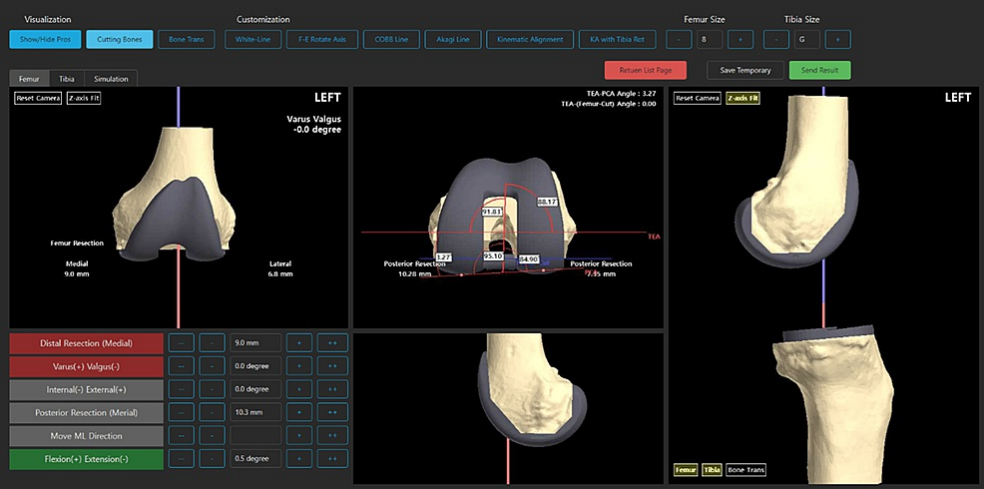
Preoperative surgical plan for TKA surgery (on web-based surgical planning software) TKA: total knee arthroplasty

To create a bone 3D model, CT imaging of a patient is typically performed, followed by 3D reconstruction. In this study, the CT imaging and 3D reconstructing steps were replaced by a 3D scan of the bone model, femur, and tibia. The 3D models of bone were obtained by 3D scanning on sawbone models. Using 3D models, anatomic landmarks were identified, and surgical planning was conducted using KNEESIGN (Skyve Co. Ltd., Seoul, South Korea) web-based surgical planning software, as shown in Figure 3. Decisions on resection depth and size, varus/valgus alignment, and positioning were then determined.

2.3.2. Intraoperation

Markers were inserted on the middle of the femur and tibia bones, respectively, positioned to face the AR system upon insertion, serving as a local coordinate system (LCS) for each part. The center and rotation information of each part's LCS was captured by the Polaris camera within a global coordinate system (GCS), enabling the conversion of LCS coordinates to GCS coordinates. The femur was rotated around the hip center to locate the center of rotation. A probe was used to obtain the coordinates of the femur's anatomical landmarks, and additional point coordinates were obtained as needed. This process was integrated with the coordinate system of the lens marker.

**Figure 4:**
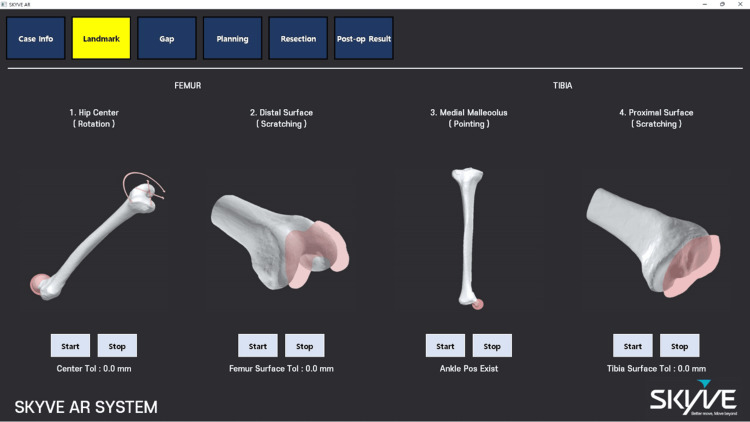
Image registration in the AR-assisted navigation system for TKA surgery AR: augmented reality; TKA: total knee arthroplasty

The discrepancy between the intended position of the 3D model and its appearance through the glasses was minimized to avoid affecting the surgery. The surface and anatomic landmark data of the bones were utilized to improve the accuracy of registration for the previously planned surgical images with the actual bone morphology. Anatomical landmarks and additional points obtained in the first stage were used to align with a pre-reconstructed 3D bone model. If the reconstructed bone was based on CT images, a technique that considered the actual bone's cartilage for alignment was necessary. The surgical planned images were registered, as shown in Figure 4.

**Figure 5:**
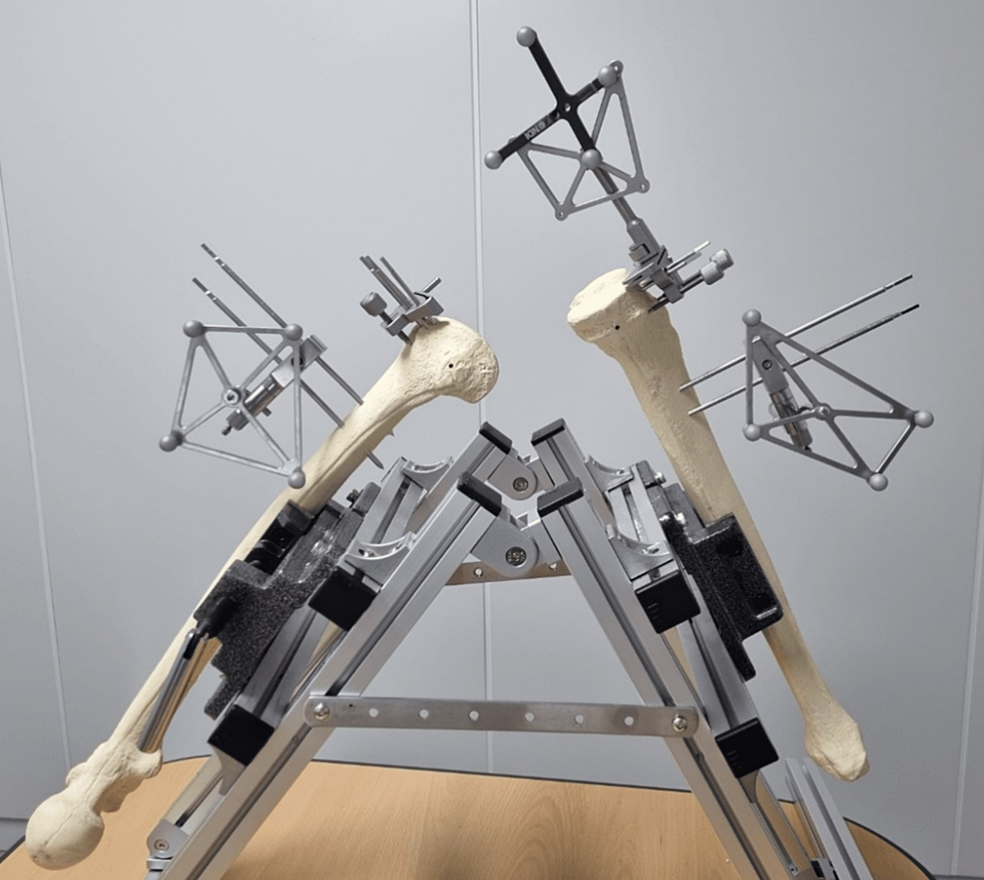
Instrument installation for the TKA AR-assisted navigation system AR: augmented reality; TKA: total knee arthroplasty

The information displayed on the glass screen was categorized into two main types: the 3D model and other information. The 3D model, including pins and implants, was implemented in AR at the desired coordinates within the coordinate system using a 3D standard tessellation language (STL) file. Other information, such as resection level, alignment angle, and size, was displayed on the screen without obstructing the view. When drilling, the actual positions were aligned with the AR-implemented instruments, and the alignment between the AR instruments and the actual instruments was verified. The instruments were installed using the AR-assisted navigation system, as shown in Figure 5, bone resection was conducted, and PNK TKA implants (Skyve Co. Ltd., Seoul, South Korea) were inserted.

**Figure 6:**
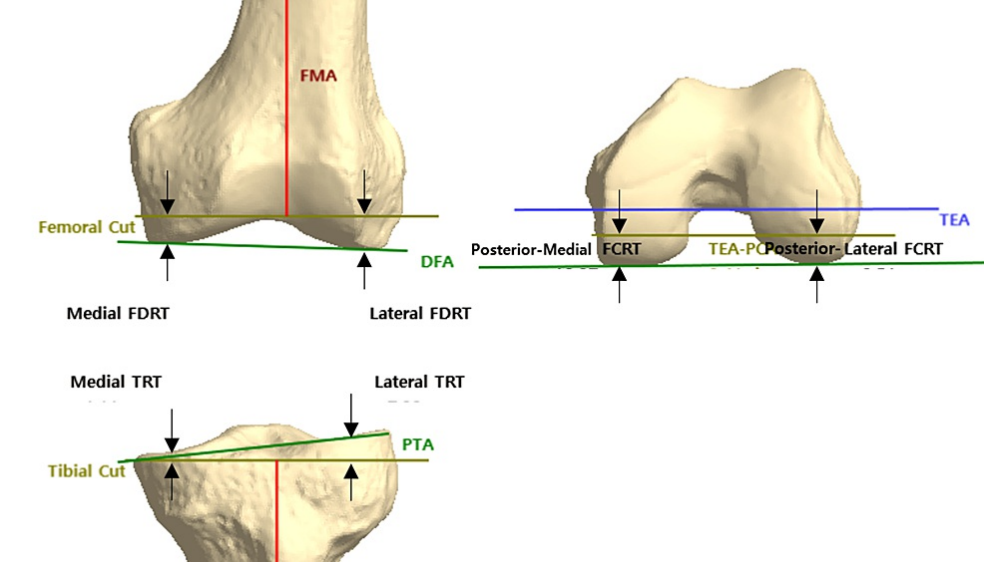
Definitions of six of the bone resection depths in TKA surgery* *Medial FDRT, lateral FDRT, posterior-medial FCRT, posterior-lateral FCRT, medial TRT, and lateral TRT FCRT: femur condyle resection thickness; FDRT: femur distal resection thickness; FMA: femoral mechanical axis; PCA: posterior condylar axis; PTA: proximal tibial angle; TEA: transepicondylar axis; TKA: total knee arthroplasty; TRT: tibia resection thickness

After conducting the bone model test, six of the bone resection depths [medial-femur distal resection thickness (FDRT), lateral-FDRT, medial-femur condyle resection thickness(FCRT), lateral-FCRT, medial-tibia resection thickness(TRT), and lateral-TRT] were assessed, as shown in Figure 6. Six of the bone resection depths in the bone model test using an AR-assisted system were compared with the expected depth in the preoperative surgical plan.

## Results

3.1. AR system validation

3.1.1. Single-Point Accuracy Test

The position was measured 20 times for point 20 (as shown in Figure 2), and the error value compared to the average value was obtained. The results of the single-point accuracy test are shown in Table 1. The error value was 0.16 mm on average, with a 0.10 mm standard deviation (SD), and a maximum value of 0.32 mm.

**Table 1:** Results of single-point accuracy test SD: standard deviation

No.	X (mm)	Y (mm)	Z (mm)	Distances of all the measurements from this average point (mm)
#1	-54.8	-84.1	8.4	0.32
#2	-54.8	-84.1	8.5	0.30
#3	-55.1	-84.0	8.5	0.02
#4	-55.3	-83.9	8.5	0.24
#5	-55.3	-83.9	8.6	0.26
#6	-55.1	-84.0	8.5	0.02
#7	-55.3	-84.1	8.4	0.25
#8	-55.1	-84.2	8.5	0.19
#9	-55.2	-83.9	8.5	0.16
#10	-55.1	-84.0	8.5	0.02
#11	-55.0	-84.1	8.5	0.12
#12	-55.3	-83.9	8.4	0.26
#13	-55.1	-84.0	8.5	0.02
#14	-55.1	-84.0	8.6	0.11
#15	-55.0	-84.0	8.6	0.14
#16	-54.9	-83.9	8.5	0.22
#17	-55.3	-84.0	8.4	0.23
#18	-54.9	-84.1	8.5	0.21
#19	-55.1	-84.1	8.5	0.09
#20	-55.0	-84.0	8.5	0.09
Average	-55.09	-84.02	8.495	0.16
SD	0.16	0.09	0.06	0.10
Maximum value				0.32

3.1.2. Point-to-Point Distance Error Test

A total of 20 points was measured, as shown in Table 2, and the distance between the points was calculated. A total of 190 distances were generated from one point to another. Errors were measured by comparing the calculated distance with the correspondence distances from the ground truth based on the phantom. The error value was 0.44 mm on average, with a 0.37 mm standard deviation, and a maximum value of 1.42 mm.

**Table 2:** Results of point-to-point distance error test

Point no.	X (mm)	Y (mm)	Z (mm)
1	-53.5	-39.1	18.5
2	-64.2	-48.7	16
3	-43.7	-49.5	16.5
10	-105.7	-87.2	6.4
11	-4.2	-90.6	8.5
18	-148.8	-125.9	-3.4
19	35.4	-131.8	0.5
22	-102.8	-127.4	-2.3
24	-57.1	-128.8	-1.3
26	-11	-130.2	-0.5
29	-97.5	-156.6	-19.3
30	-77.4	-150.3	-17.3
32	-37	-150.5	-21.3
33	-17.3	-158.1	-22.4
36	-56.5	-166.8	-48.2
40	-88.4	-175.8	-64.6
41	-56.7	-176.8	-63.9
42	-24.3	-177.8	-63.1
46	-65.6	-198.4	-74.9
47	-49.5	-199	-74.6

3.2. Bone resection test using AR assistance in TKA surgery

The results of the bone resection test were analyzed, as outlined in Table 3.

**Table 3:** Bone resection depths and errors in bone resection depths in the preoperative surgical plan, AR-assisted navigation system, and bone model test AR: augmented reality; FCRT: femur condyle resection thickness; FDRT: femur distal resection thickness; SD: standard deviation; TRT: tibia resection thickness

	Bone resections in			Errors between		
	① Preoperative surgical plan	② AR-assisted navigation system	③ Bone model test	① & ②	① & ③	② & ③
Medial FDRT (mm)	9	8.9	9.1	0.1	0.1	0.2
Lateral FDRT (mm)	10.84	10.6	10.7	0.24	0.14	0.1
Posterior-medial FCRT (mm)	10.07	9.9	9.6	0.17	0.47	0.3
Posterior-lateral FCRT (mm)	9.51	9.3	9.1	0.21	0.41	0.2
Medial TRT (mm)	1.11	1.3	1.5	0.19	0.39	0.2
Lateral TRT (mm)	7	7.2	7.4	0.2	0.4	0.2
Average				0.19	0.32	0.2
SD				0.043	0.143	0.058

Using the AR-assisted navigation system, the instrument was installed according to the values determined by the preoperative surgical plan. The average error between bone resection depths in the preoperative surgical plan and AR-assisted navigation system was 0.19 mm, with an SD of 0.043 mm. The average error between the bone resection depths in the preoperative surgical plan and bone test model was 0.32 mm, with an SD of 0.143 mm. The average error between bone resection depths in the AR-assisted navigation system and bone test model was 0.2 mm, with an SD of 0.058 mm.

## Discussion

The most important finding of this study was that image-based AR in TKA surgery achieved precise bone resection. In this study, AR-assisted surgery using the proposed image method showed fewer errors (average error 0.32 mm; SD: 0.143) compared to AR-assisted surgery using the imageless method (average error: 0.6 mm; SD: 0.7 mm) [11]. In the medial FDRT, lateral FDRT, posterior-medial FCRT, posterior-lateral FCRT, medial TRT, and lateral TRT, the errors in the bone model test compared to the preoperative surgical plan were as follows: 0.1, 0.14, 0.47, 0.41, 0.39, and 0.4 mm, respectively. These results correspond to 16.7%, 23.3%, 78.3%, 68.3%, 65%, and 66.7% respectively, when compared to the error of 0.6 mm reported in the previous study using imageless technology-based AR-assisted surgery [11]. Therefore, it appears that AR-assisted surgery utilizing the proposed image method exhibits higher levels of accuracy in comparison to AR-assisted surgery employing the imageless method. The results of previous studies highlight the strengths of our research. Our findings suggest that, regarding the application of AR technology in TKA surgery, image-based technology may be more suitable than imageless technology.

Various techniques of computer-assisted surgical navigation have been developed over the last few years to enhance accuracy and precision in component positioning [15]. An overall decrease in revision rate has been reported in TKA when utilizing computer-assisted surgery [16]. Moreover, various technological innovations have been incorporated into orthopedic surgery, including 3D-printed patient-specific instrumentation (PSI) [4], navigation tools with visualized tracking on monitors [3], and robotic surgery [17]. PSI has proven to be a viable tool with excellent postoperative results [18,19]. Nevertheless, the design and manufacturing of PSI require several weeks of preparation time [20]. Furthermore, the need for extensive bone exposure in the execution of navigated surgical procedures is a drawback [21]. Robotic surgery yields favorable clinical outcomes; however, it has certain limitations in broader application, and the expenses associated with robotic surgery systems are considerably high and can exceed USD one million [5]. Moreover, robotic surgery systems are space-consuming, and their setup and maintenance are time-consuming and cost-intensive, adding an average of 10-15 min per case and imposing a lengthy learning curve [4].

The recently introduced AR technology is expected to enhance TKA accuracy and is regarded as a more efficient and cost-effective solution compared to robotic-assisted surgery [5]. While robotic technologies primarily aim to assist surgeons with precise and planned mechanical actions, technologies such as AR can enhance the surgeon's ability through intuitive augmentation of medical information [8]. AR is characterized as a technology that enhances the real world with virtual information through the utilization of smart glasses worn by the surgeon [9]. Thus, AR possesses the ability to overlay the intended shape of a 3D digital simulated image onto the patient's knee during surgery. This capability is especially crucial for knee replacement procedures, where accurately identifying cartilage conditions and cutting angles for precise placement of specific artificial components are paramount [22]. Given the ongoing shift towards personalized surgery, AR surgery appears to be an innovative technology with considerable potential for use in modern artificial joint procedures.

Robotic surgery has emerged as a prominent computer-assisted surgical approach, where both image-based knee arthroplasty and imageless knee arthroplasty surgeries are performed. Previous studies have compared operative time, intraoperative blood loss, and radiologic and clinical outcomes between imageless NAVIO (Journey UNI Unicompartmental Knee System, Smith & Nephew, Memphis, TN) and image-based MAKO (Restoris MCK Partial Knee Implant System, Stryker, Kalamazoo, MI) robot-assisted unicompartmental knee arthroplasty (UKA) for medial compartment osteoarthritis (OA) of the knee [23]. Their study showed that the operative time and intraoperative blood loss were lower in image-based MAKO robot-assisted UKA compared to imageless (NAVIO) robot-assisted UKA [23]. Recently, Vermue et al. demonstrated that the imageless method lacks the precision and repeatability of image-based technology [24]. Currently, only imageless products such as Knee+ (Pixee Medical Company, Besancon, France), ARvis (Enovis, CA), and Polaris AR (Polaris AR, Miami, FL) are commercially available, not image-based products such as Sagarvision AR.

The main advantage of imageless methods over image-based ones is that they do not require preoperative CT imaging. However, an increased surgical time is required. Both image-based technology and imageless methods have demonstrated excellent accuracy in coronal alignment [24]. Nonetheless, recent studies have shown that the imageless method lacks the precision and repeatability required for defining the sagittal plane of the tibia compared to image-based technology [24]. Additionally, there is a lack of scientific evidence supporting an accurate and precise definition of the sagittal plane of the tibia in imageless technology [24]. A previous study has highlighted notable variations in the surgeons' intraoperative proficiency in identifying anatomical landmarks on the proximal tibia, with a specific emphasis on the tibial tuberosity [25]. The AR-assisted approach offers the advantage of a shorter learning curve and mitigates the impact of surgeon proficiency variability in surgical procedures. From this perspective, AR-assisted systems hold the potential to complement conventional instrumentation. To the best of our knowledge, our study is the first of its kind to assess TKA performed under the guidance of image-based AR technology as opposed to conventional imageless methods. Our results suggest that, in TKA surgery, image-based methods may offer better efficiency compared to imageless methods when utilizing AR technology. Additionally, image-based AR technology shows the potential to assist surgeons in performing TKA surgery with enhanced precision and accuracy, potentially leading to reduced operative time and radiation exposure.

Our study has some limitations. The materials used were sawbones without soft tissue. The precision achieved by touching bony landmarks through soft tissues was influenced by the thickness of the soft tissue [26]. Therefore, further research targeting patients is necessary. We have consistently highlighted the advantages of image-based approaches. However, the system requires the assistance of a dedicated clinical engineer to generate a 3D model of the patient's anatomy based on medical imaging data. This study did not involve a human participant as a patient but used the sawbone model in a laboratory environment. In addition, the lack of a direct comparison with the imageless method could be a potential limitation.

## Conclusions

Our findings highlight the accuracy of bone resection in image-based AR TKA surgery. This technique is not only a valuable method for enhancing accuracy by using smart glasses and sensors but also improves the efficiency of the procedure. Unlike the imageless approach, image-based AR in TKA surgery leverages the patient's anatomical data for preplanning the surgery in three dimensions, ensuring accurate bone resection in both coronal and sagittal planes. Therefore, we anticipate that image-based AR in TKA surgery will be widely used in everyday practice.
